# A compressive seeding algorithm in conjunction with reordering-based compression

**DOI:** 10.1093/bioinformatics/btae100

**Published:** 2024-02-20

**Authors:** Fahu Ji, Qian Zhou, Jue Ruan, Zexuan Zhu, Xianming Liu

**Affiliations:** School of Computer Science and Technology, Harbin Institute of Technology, Nan Gang District, Harbin 150080, China; Peng Cheng Laboratory, Nanshan District, Shenzhen 518055, China; Shenzhen Branch, Guangdong Laboratory of Lingnan Modern Agriculture, Genome Analysis Laboratory of the Ministry of Agriculture and Rural Affairs, Agricultural Genomics Institute at Shenzhen, Chinese Academy of Agricultural Sciences, Dapeng District, Shenzhen 518120, China; College of Computer Science and Software Engineering, Shenzhen University, Nanshan District, Shenzhen 518060, China; School of Computer Science and Technology, Harbin Institute of Technology, Nan Gang District, Harbin 150080, China; Peng Cheng Laboratory, Nanshan District, Shenzhen 518055, China

## Abstract

**Motivation:**

Seeding is a rate-limiting stage in sequence alignment for next-generation sequencing reads. The existing optimization algorithms typically utilize hardware and machine-learning techniques to accelerate seeding. However, an efficient solution provided by professional next-generation sequencing compressors has been largely overlooked by far. In addition to achieving remarkable compression ratios by reordering reads, these compressors provide valuable insights for downstream alignment that reveal the repetitive computations accounting for more than 50% of seeding procedure in commonly used short read aligner BWA-MEM at typical sequencing coverage. Nevertheless, the exploited redundancy information is not fully realized or utilized.

**Results:**

In this study, we present a compressive seeding algorithm, named CompSeed, to fill the gap. CompSeed, in collaboration with the existing reordering-based compression tools, finishes the BWA-MEM seeding process in about half the time by caching all intermediate seeding results in compact trie structures to directly answer repetitive inquiries that frequently cause random memory accesses. Furthermore, CompSeed demonstrates better performance as sequencing coverage increases, as it focuses solely on the small informative portion of sequencing reads after compression. The innovative strategy highlights the promising potential of integrating sequence compression and alignment to tackle the ever-growing volume of sequencing data.

**Availability and implementation:**

CompSeed is available at https://github.com/i-xiaohu/CompSeed.

## 1 Introduction

As of today, next-generation sequencing (NGS) technologies have been ubiquitous and widely used in academic and industry areas. One of the most prominent characteristics of NGS platforms is that they produce large volumes of short and accurate fragments of the sequenced genome, known as reads. Over the past two decades, a suite of computational tools has been fully developed to efficiently handle the high-throughput data. For example, there are hundreds of optimization algorithms to improve the performance of read alignment stage (Wilton and Szalay 2022), the first step and often the major bottleneck in genomic pipelines (Van der Auwera *et al.* 2013). Nevertheless, the demand for faster data analysis remains ongoing as the number of available sequencing reads continues to exponentially increase.

BWA-MEM (Li 2013), one of the most popular read aligners for NGS reads, has been accelerated by various solutions, which are typically based on hardware optimization because the identical alignment result is required to guarantee the reproducibility of genomic analysis experiments. In this background, BWA-MEM has been deployed in GPU platforms (Houtgast *et al.* 2016), FPGA chips (Houtgast *et al.* 2015), and distributed clusters (Vasimuddin *et al.* 2019) after necessary reconstruction and modifications. Like other classical aligners, BWA-MEM follows the seed-and-extend paradigm, where seeding determines the maximal exact matches (MEMs) between the read and the reference and extension performs dynamic programming around the MEMs to complete the alignment. Either seeding or extension contributes about half of BWA-MEM running time. Since seeding and extension are two individual sequential processes, they can be treated as separate tasks when improving the read alignment.

For the extension stage, parallelized instructions can elegantly solve the computation-intense problem, such as SIMD instructions (Farrar 2007) and GPU architectures (Liu *et al.* 2010), which commonly accelerate the regular Smith–Waterman algorithm dozens of times and can be easily integrated to the existing optimized versions of BWA-MEM. However, the solutions for speeding up seeding stage are fewer in comparison to the extension stage. This is mainly because the random access mechanism of FM-index (Ferragina and Manzini 2000) renders the hardware optimization difficult. Similar problem exists in other FM-index-based aligners, such as Bowtie2 (Langmead and Salzberg 2012), Gem (Marco-Sola *et al.* 2012), and Kart (Lin and Hsu 2017). With the advances in hardware optimizations of the extension stage, the bottleneck of seeding becomes more prominent and inspires researchers to put more efforts in the seeding stage.

The existing approaches modify or replace the FM-index to accelerate seeding, because the original FM-index significantly limits the capability of hardware and restricts the space for improvement. First, both BWT data structure and suffix array are sparsely stored (Li and Durbin 2009), which leads to a large constant for each BWT operation and suffix array lookup (SAL). Second, frequent random accesses to memory are cache unfriendly and lead to poor spatial locality. BWA-MEM2 (Vasimuddin *et al.* 2019) uses a smaller sampling interval for storing BWT data structure and suffix array that introduces a four times memory overhead, but it is still limited by the data prefetch bound. BWA-MEM2 next adopts enumerated radix tree (ERT) (Subramaniyan *et al.* 2021) to entirely replace the FM-index, and performs the super maximal exact match (SMEM) search algorithm (Li 2012) in a zigzag fashion to reduce the unsuccessful SMEM extensions. By encoding the suffixes of the reference sequence in a radix tree, ERT significantly improves the space locality of seeding at a cost of 64 GB memory for human genome index.

Recently, solutions based on machine learning are proposed. An early study (Kraska *et al.* 2018) has proved that machine learning can utilize the imbalance distribution within data to accelerate traditional indices, such as B-Tree. Sapling (Kirsche *et al.* 2021) implements a learned index structure for suffix array. It encodes a query string to a number and through an artificial neural network to predict the matched locations in the suffix array. The prediction is bounded by an error limit, restricting the binary search within a small range of suffix array. While Sapling primarily supports fixed-length seeding, it is suitable for Bowtie2 but not BWA-MEM. LISA (Ho *et al.* 2020) employs a learned data structure called IP-BWT, enabling a single lookup to match 21 bases and reducing the times of memory access. Subsequently, BWA-MEME (Jung and Han 2022) utilizes a Partially Recursive Model Index model to further address the imbalanced distribution of *k*-mers in the network, and exhaustively reimplements BWA-MEM seeding to enhance the cache utilization. BWA-MEME notably outperforms ERT with the cost of 110 GB RAM for human sequencing data.

In this study, we propose an approach to accelerate seeding distinct from all the above methods. Our algorithm utilizes the redundancy information within NGS reads to remove the repetitive FM-index operations during the BWA-MEM seeding procedure. Similar idea has been proposed in previous works, but they mainly focus on the redundancy within reference genomes. CompMap (Zhu *et al.* 2015) compresses the related genomes to complete tasks like pathogen discovery and metagenomic classification with a significantly reduced time and memory usage. CORA (Yorukoglu *et al.* 2016) generates a high-resolution homology table across the reference genome to rapidly locate the repetitive alignments, which are enriched for human genomes. deBGA (Liu *et al.* 2016) organizes and indexes a set of reference sequences with de Bruijn Graph that naturally captures the repetitive regions in a genome and the homology regions between genomes. deBGA servers as an NGS aligner for pangenome projects as it fully utilizes the redundancy from the reference end (Wang *et al.* 2022). However, the significant redundancy within sequencing data has not received sufficient attention. One possible reason is that it is a non-trivial challenge to fully utilize the redundancy to accelerate alignment without loss in sensitivity and accuracy. For example, CORA extracts the representative *k*-mers to “compress” reads and leads to a maximum sensitivity loss of ∼1.2% in best alignment mode.

Our study aims to fill this gap. We propose the algorithm, namely CompSeed, that completes the BWA-MEM seeding process in half the time by utilizing the prior knowledge from upstream data compression. We do not develop a specific compression algorithm because there is a slew of specialized compression tools that are full-fledged and push the compression ratio to the limit (Hernaez *et al.* 2019). These tailored compressors are promoted by the emerging compression standards like MPEG-G and AVS-G to replace the general-purpose tools, such as gzip, which is widely used but with a much worse compression ratio. Among the abundant choices, we employ the reordering-based compression tools as they have been proven to achieve superior compression rates. Reordering-based compressors including SPRING (Chandak *et al.* 2019), Minicom (Liu *et al.* 2019), PgRC (Kowalski and Grabowski 2020), Mstcom (Liu and Li 2021), and CURC (Xie *et al.* 2022) adopt various strategies to identify overlaps between reads and approximately rearrange them based on their derived positions in the genome. Usually, the original order of reads is discarded because maintaining it significantly degrades the compression ratio and is not necessary for downstream analysis. Instead, the reordering layout of reads is much more meaningful than the random order in FASTQ (Cock *et al.* 2010) format, though it is overlooked or underutilized.

The key insight is that the reordering of reads explicitly brings out the inherent data redundancy by clustering similar reads together, which are randomly distributed in FASTQ files, and the repetitive computations during seeding can be avoided by reusing the intermedia results of preceding reads. In fact, the seed redundancy within reordered reads is substantial. We found that over half of BWT and SAL operations are identical across the entire BWA-MEM seeding process at common sequencing coverage. This observation provides an opportunity to accelerate the traditional seeding without modifying or replacing the FM-index. Receiving the compressed results from reordering-based compression tools, CompSeed effectively merges a large number of redundant FM-index operations between neighboring reads by storing the preceding results in compact trie structures. As a result, CompSeed reduces the time cost of BWA-MEM seeding by about half without increasing memory footprint. After accelerating Smith–Waterman stage with SIMD instructions, CompSeed demonstrates its cost-effective practicability compared with the existing solutions.

The potential of using data compression to enhance computational analyses has been proved in early works, where the authors proposed the compressive versions (Loh *et al.* 2012) of BLAST (Altschul *et al.* 1997) and BLAT (Kent 2002), and analyzed the theoretical advantage of compression in prior analysis (Berger *et al.* 2016). They advocate that focusing only on the non-redundant part is a promising approach for handling NGS data, because the valuable information grows much slower than the whole exponentially increasing data volume. The accomplishment of CompSeed demonstrates the practicality of compressive mode again. Additionally, by fully exploiting the information in compressed data, CompSeed performs better when the compression is optimized or the sequencing coverage increases, making it more suitable for advanced NGS technologies that produce sequencing data in higher quality and larger quantity.

## 2 Materials and methods

Before moving on to the compressive seeding algorithm, we first provide a brief description of the FM-index and BWA-MEM seeding algorithm. For more detailed information, readers can refer to Ferragina and Manzini (2000) and Li (2013). To obtain all matching locations of a substring of a read in the reference sequence using FM-index, two steps are required. First, the query string is extended one character at a time in either direction using a recursion process called BWT-extend, which determines the suffix array interval (SAI) of *aS* or *Sa* based on the SAI of string *S*, where *a* is an added character. Second, once the SAI is obtained, all occurrences of the query string in reference genome can be located by accessing the corresponding range of the suffix array. The size of SAI is an important value in seeding because it indicates the end of BWT-extend and allows for the skipping of repetitive seeds. For clarity, we define an “exact match” of a query string as its SAI, which is obtained by BWT-extend and represents the relationship between the query string and the suffix array. A “seed” represents one occurrence position of the exact match in the reference genome and is obtained using a SAL operation. An exact match might correspond multiple seeds depending on the SAI size. During the seeding process, both BWT-extend and SAL are frequently called to produce sensitive seeds for accurate alignment. However, both of them are time-consuming functions due to sparely stored data structure and random memory accesses.

**Figure 1.**
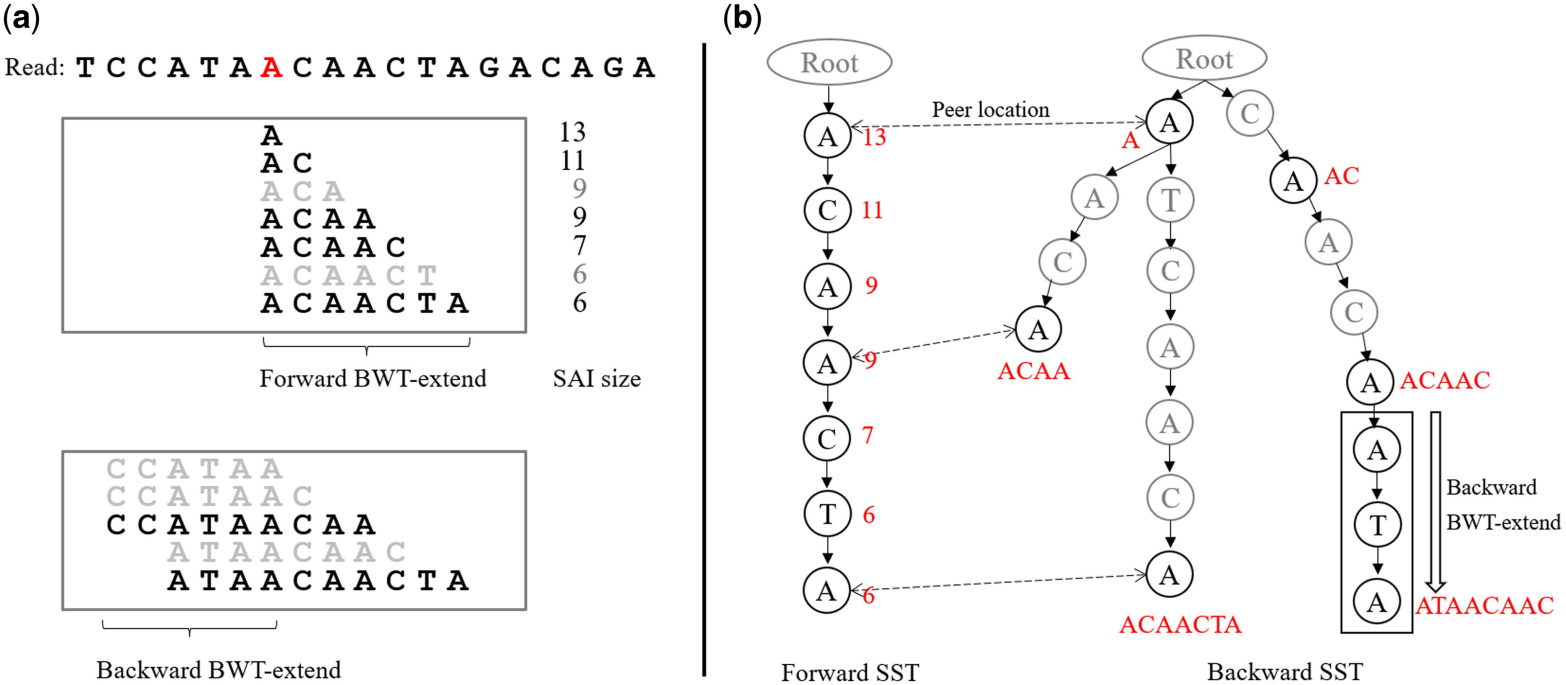
Illustration of SuperMEM1 and SST. (a) During the forward search stage of SuperMEM1 algorithm, exact matches are forwardly extended from the pivot base (highlighted in red) and are recorded when the SAI size changes. These exact matches are then backwardly extended to the limit, and those not contained within other matches are selected as SMEMs. (b) All the intermedia results of forward and backward stages of SuperMEM1 are stored in the forward and backward SST, respectively. A node in the forward SST stores the SAI of an exact match spelling from the root node to the current node. The root node is only used to compact the trie and carries no character. The exact matches to backwardly extend are inserted into the backward SST character by character in reverse order. The bidirectional dashed arrows show the peer locations of the exact matches. This process introduces some empty nodes with unknown SAI, which are shown in grey color in the figure and marked with SAI size = −1 in the implementation.

BWA-MEM seeding chooses the SMEMs to locate candidate alignments. A SMEM is an exact match that cannot be extended in both directions under a certain SAI size threshold *min-intv*. Firstly, BWA-MEM collects all SMEMs across the read with the *min-intv* set to one. Secondly, in case, the optimal alignment does not contain any such SMEM, BWA-MEM introduces re-seeding to collect the SMEMs with an increased *min-intv* to cover the middle base of those ultra-long SMEMs. Both stages use the same function SuperMEM1(*pivot*, *min-intv*) in Algorithm 5 from Li (2012), which returns all SMEMs covering the *pivot* position in the query read with the SAI size not smaller than *min-intv*. We borrow the example from Jung and Han (2022) to illustrate the SuperMEM1 algorithm (Fig. 1a), but not expound it because we do not reshape the algorithm.

SuperMEM1 comprises two searching stages: forward search and backward search. The forward search retains all exact matches with a changed SAI size, starting from the pivot base. The reason is that if two exact matches share a same prefix and have a same SAI size, the shorter match certainly cannot be a SMEM. The forward search continues extending the last exact match using the forward BWT-extend operations and stops until the SAI size <*min-intv* or reaching the end of the read. The backward search extends the retained exact matches to obtain the final SMEMs using the backward BWT-extend operations. Both ERT and BWA-MEME algorithms reconstruct the SuperMEM1 function for their specific index structures, providing faster unit operations than the original BWT-extend and SAL. However, our CompSeed has not. We achieve speedup by reducing the redundant accesses to the FM-index that are explicitly brought out by upstream compressors.

Although the reordering-based compressors adopt different approaches, their common goal is to identity the similarity between reads as much as possible. They finally provide a reordered layout where consecutive reads typically share a large overlap with each other. Based on our observation, more than half of computations in such overlaps are redundant when seeding is sequentially performed. For BWT-extend operations, exact matches in the overlapping part of neighboring reads might query same substrings. It gives a chance that subsequent exact matches can directly reuse the results of previous read, avoiding the time-consuming access to FM-index. The redundancy is more obvious for SAL operations. The repetitive lookups to suffix array come from the intersected SAI of exact matches. They can be easily identified and merged. The major challenge is to efficiently store and reuse the previous results of BWT-extend. It is crucial to pick an efficient data structure to prevent the overhead from canceling the benefits of reusing.

In order to address this problem, we introduce a trie structure called SMEM Search Trie (SST), which follows the same rule as a word retrieval tree, but with some minor modifications. To align with the structure of SuperMEM1, we create both forward and backward SST for storing the results of forward and backward BWT-extend, respectively. Figure 1b illustrates the process of building SST. The forward SST is straightforward, with each node storing the SAI of an exact match that spells from the root node (an empty node with no character) to the current node. Each downward edge represents a forward BWT-extend, allowing the results of all emerging prefixes to be compactly stored in the trie. The backward SST is a bit more complicated. As the successor of the forward SST, the backward SST receives the exact matches to be backwardly extended. However, those exact matches cannot be represented as an independent node in a trie. To handle this, we break down an exact match into single characters and add them to the backward SST in reverse order. As shown in Fig. 1b, an exact match will first find its peer location in the backward SST before further backward BWT-extend. This process introduces some empty nodes that we do not know the SAI. In the illustrated example, we lack the SAI information of “CAA” and “AA” when adding “ACAA” to the backward SST. We mark those nodes empty with SAI size =−1. They will be filled only if backward BWT-extend is requested on those nodes later.

**Figure 2.**
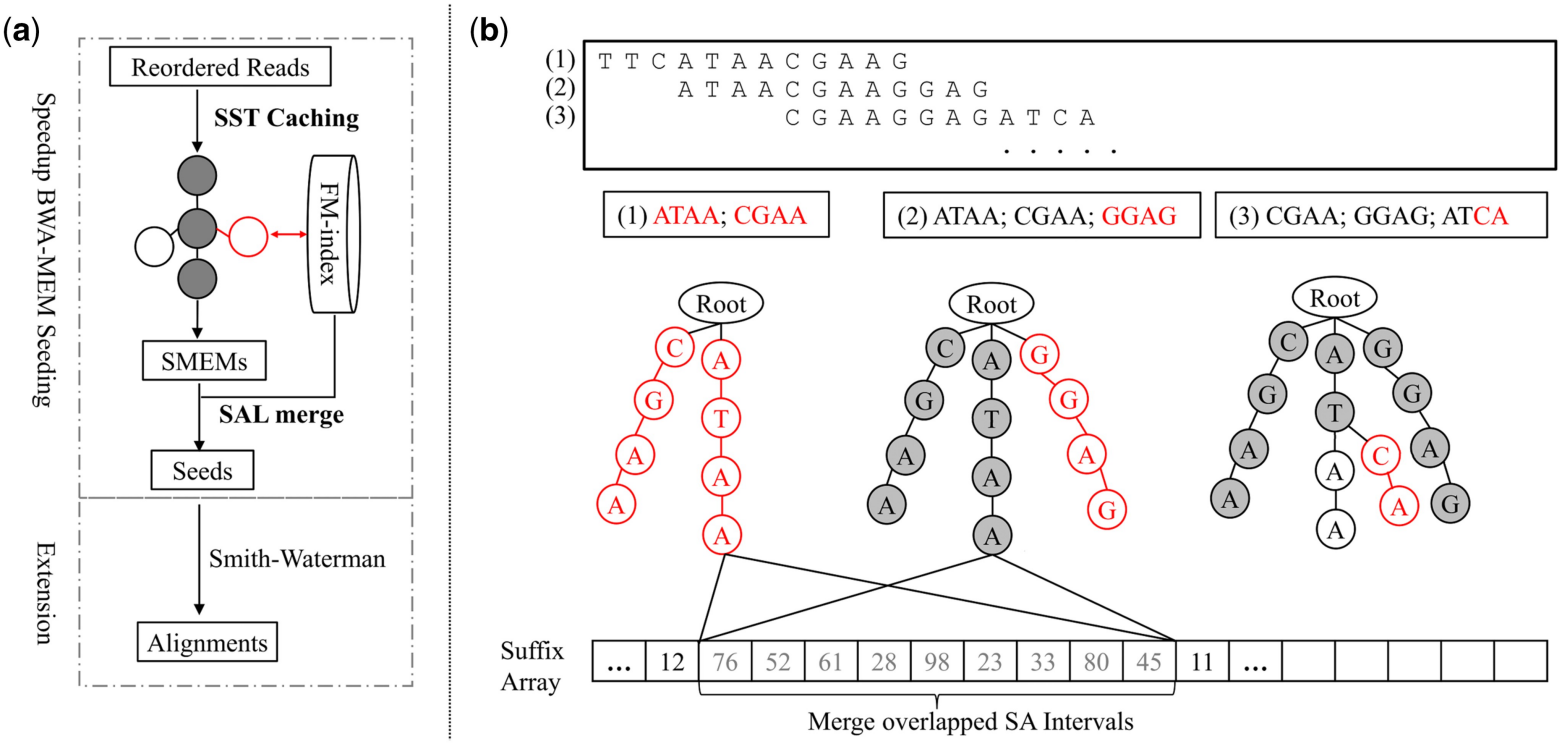
(a) Schematic workflow of CompSeed. CompSeed follows the same procedure as BWA-MEM, except for the addition of SST data structure to cache the intermedia results in seeding stage. The SST only accesses FM-index when the queried seed (marked in red circle) is absent from the tries, whereras BWA-MEM always accesses FM-index regardless of redundancy. (b) An example of processing three reads. The SST grows with the number of processed reads, along with an increased chance to directly answer repetitive queries (shadowed nodes). In the end, the SALs for exact matches with identical or overlapped SAI are merged.

In this way, we move all the computations in SuperMEM1 to the SST. When processing a batch of reads, each request of BWT-extend will first inquiry the SST. If the corresponding node is present in the SST, the result is directly returned. Otherwise, SST accesses the FM-index to obtain the result that a new node is added to store for future inquiries. As more reads are processed, the SST grows in size, and an increasing number of BWT-extend requests are directly answered with the cached results in the SST. In the same time, to prevent the SST from expanding excessively, we set the batch size as 512. The batch size is chosen as it contains sufficient data redundancy. The overall process is shown by Fig. 2.

The code implementation of CompSeed is concise. We replace the BWT-extend function call in SuperMEM1 with the interface provided by SST, which acts as an intermediary and replies the BWT-extend inquiry with only necessary accesses to FM-index. Moreover, CompSeed could work with any reordering-based compressor. Our design directly takes the reordered reads as input to avoid the huge efforts to support various compression formats. While it is worth noting that being compatible with compression formats could be beneficial for compressive seeding, because it does not need explicit decompression and provides valuable information, such as corrected strand of reads and offsets between matched reads.

## 3 Results

We compared the time cost of CompSeed and BWA-MEM seeding and alignment time on eight datasets, with typical sequencing coverage and varied-scale genomes (Supplementary Tables S1–S3). All the experiments were carried out on a machine equipped with Intel(R) Xeon(R) Gold 6242 CPU @ 2.80 GHz with 20 cores, 256 GB of RAM, and the CentOS (release 7.4.1708) Linux distribution. BWA-MEM worked on the original FASTQ files while CompSeed on the reordered reads that were compressed and subsequently decompressed by three classical compressors, namely SPRING, Minicom, and PgRC. The resource consumption of data compression and decompression are provided in Supplementary Tables S4–S8. These professional tools have comparable speed but much better compression ratio compared with commonly used gzip. All experiments were run in 16 threads, and the runtime was recorded by hand instrumenting trackers into the source code.

Table 1 lists the sizes of original bases and compressed files, as well as the time cost of seeding performed by BWA-MEM and CompSeed on them. CompSeed works on much smaller compressed files, and outperforms BWA-MEM by completing the same seeding process in about half the time. CompSeed shows better performance on SPRING and PgRC than on Minicom. It suggests that SPRING and PgRC achieve a better compression ratio by more efficiently exploiting data redundancy, which allows CompSeed to merge more repetitive seeding operations. Though the difference is not significant for CompSeed. In summary, the results demonstrate that compression can lead to reduced storage requirements and seeding computations simultaneously. The worth of compression is beyond of smaller file sizes themselves. Besides, the seeding speedup offered by CompSeed can be easily combined with the current Smith–Waterman optimization to enhance end-to-end alignment performance. We used the AVX instructions that are available on common servers and obtained a doubled alignment throughput over BWA-MEM, also without introducing additional memory overhead. It is worth noting that the postprocessing of CompSeed, such as mapping quality calculation and CIGAR generation, remain unchanged from those of BWA-MEM, ensuring the identical BAM alignments.

**Table 1.** The file size, seeding, and alignment time for the uncompressed and compressed reads.

Organism	Accession No.	File size (B)				Seeding time (s)				Alignment time (s)			
		Original Bases	Compressed by			BWA-MEM	CompSeed with			BWA-MEM	CompSeed with		
			SPRING	Minicom	PgRC		SPRING	Minicom	PgRC		SPRING	Minicom	PgRC
*E*.*coli*	SRR1562082	588M	6.2M	6.7M	5.1M	165	47	54	45	217	95	104	95
*C*.*elegans*	SRR16905161	2.3G	74M	79M	55M	1529	653	629	676	3488	1765	1764	1778
*G*.*gallus*	SRR13537343	25G	563M	749M	521M	28 849	11 069	12 210	11 751	59 982	30 518	31 690	30 996
*H*.*sapiens*	ERP001775	87G	1.7G	2.2G	1.5G	130 725	51 799	58 645	54 721	205 400	96 921	103 880	99 756
*H*.*sapiens*	ERR194146	82G	2.2G	2.2G	1.5G	121 849	56 559	61 128	57 328	193 975	100 883	105 642	101 356
*H*.*sapiens*	ERR194161	85G	2.0G	2.2G	1.4G	122 252	55 409	59 951	55 289	191 997	98 977	103 804	98 315
*H*.*sapiens*	ERR3239279	63G	1.4G	1.8G	1.2G	86 982	44 127	48 967	47 038	140 462	77 666	82 751	80 329
*H*.*sapiens*	SRR10965089	56G	1.6G	2.1G	1.5G	98 605	50 932	55 461	54 679	154 289	91 278	96 807	94 923

As previously mentioned, CompSeed achieves speed gains by reducing the number of times it accesses the FM-index, specifically the BWT-extend and SAL functions, which are both time-consuming and frequently called during seeding. As shown in Table 2, CompSeed merged over half the repetitive BWT-extend and SAL calls in BWA-MEM seeding. The reduction in the number of FM-index operations basically falls in line with that in the seeding time cost observed in Table 1. It suggests that the SST structure efficiently avoids the redundant FM-index operations without introducing significant overhead. The table presents the average number of FM-index operations per read. Notably, when handling reads with longer sequencing length or more complex genomes, the computations increase as more seeds are generated. Across all datasets, CompSeed robustly reduces the number of FM-index operations and translates to faster processing time.

**Table 2.** The average call times of BWT-extend and SAL per read.

Accession No.	Read length	BWT-extend/read				SAL/read			
		BWA-MEM	CompSeed with			BWA-MEM	CompSeed with		
		Seeding	SPRING	Minicom	PgRC	Seeding	SPRING	Minicom	PgRC
SRR1562082	150	314	103	113	100	6.54	1.72	2.20	1.66
SRR16905161	125	716	269	264	261	22.09	8.88	8.03	8.42
SRR13537343	150	712	277	294	282	28.97	9.59	10.07	9.95
ERP001775	101	619	233	248	233	29.00	10.54	11.48	10.72
ERR194146	101	629	249	257	243	29.62	11.47	12.08	11.39
ERR194161	101	613	238	247	232	28.71	10.81	11.45	10.69
ERR3239279	150	900	367	385	374	38.75	16.00	17.18	16.39
SRR10965089	151	998	466	487	483	40.92	19.63	20.83	20.53

**Figure 3.**
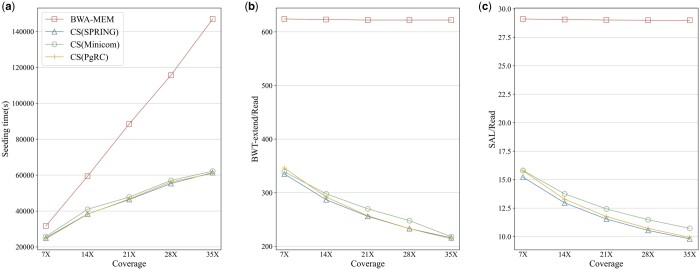
The seeding runtime (a), averaged BWT-extend (b), and SAL (c) call times each read for BWA-MEM seeding and CompSeed. CompSeed (CS for short) processes data compressed by SPRING, Minicom, and PgRC.

The seeding redundancy is proportional to the inherent data redundancy. We compared BWA-MEM seeding and CompSeed on the dataset ERP001775 with different coverage. CompSeed shows better performance with the increasing coverage (Fig. 3). This is attributed to the sublinear time complexity of compressive seeding. The basic idea is that the non-redundant, most informative part of data does not increase linearly with the amount of data. BWA-MEM seeding treats reads independently, resulting in a linear increase in runtime with the number of reads. In contrast, CompSeed takes the redundancy between reads into consideration. With more redundancy information identified, the average number of FM-index operations is actually reduced as the sequencing coverage increases. Compared to the linear traditional seeding, CompSeed is promising for processing large-scale genomic datasets, especially as today the sequencing coverage of NGS technologies continues increasing dramatically.

**Figure 4.**
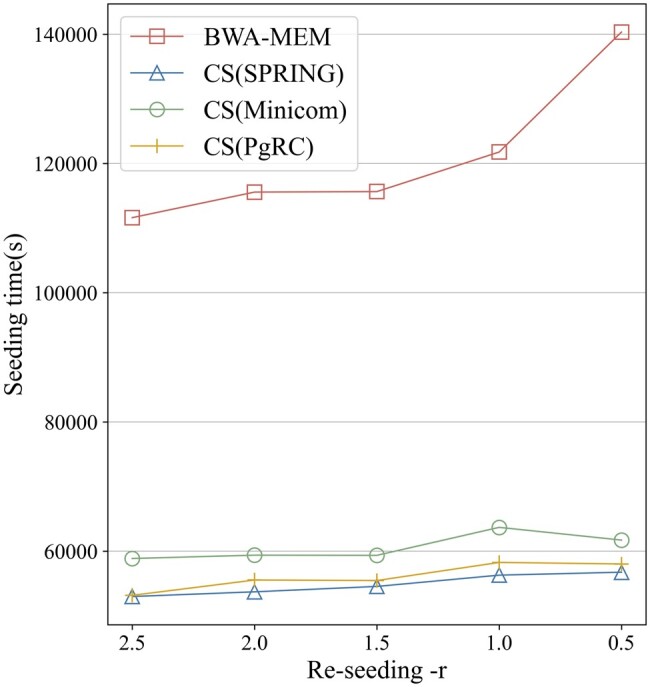
Varying the re-seeding parameter −*r*. We show −*r* in decreased order because a smaller value loosens the re-seeding condition and allows more seeds to be generated. CS is short for CompSeed.

Another prominent factor that influences seed redundancy is the re-seeding parameters −*r* (1.5 by default). When a SMEM exceeds the minimal seed length multiplied by −*r*, re-seeding is triggered to compensate potential missing optimal seeds. By choosing a smaller re-seeding parameter, more sensitive seeds can be obtained to improve the alignment accuracy (Darby *et al.* 2020). With no doubt, it also has an effect on seeding redundancy and the time cost. Figure 4 shows that the time cost of BWA-MEM seeding increases sharply when the re-seeding condition is relaxed. However, CompSeed is almost unaffected by the parameter. This is due to CompSeed’s ability to merge repetitive seeds, allowing it to perform robustly in sensitive seeding mode.

**Figure 5.**
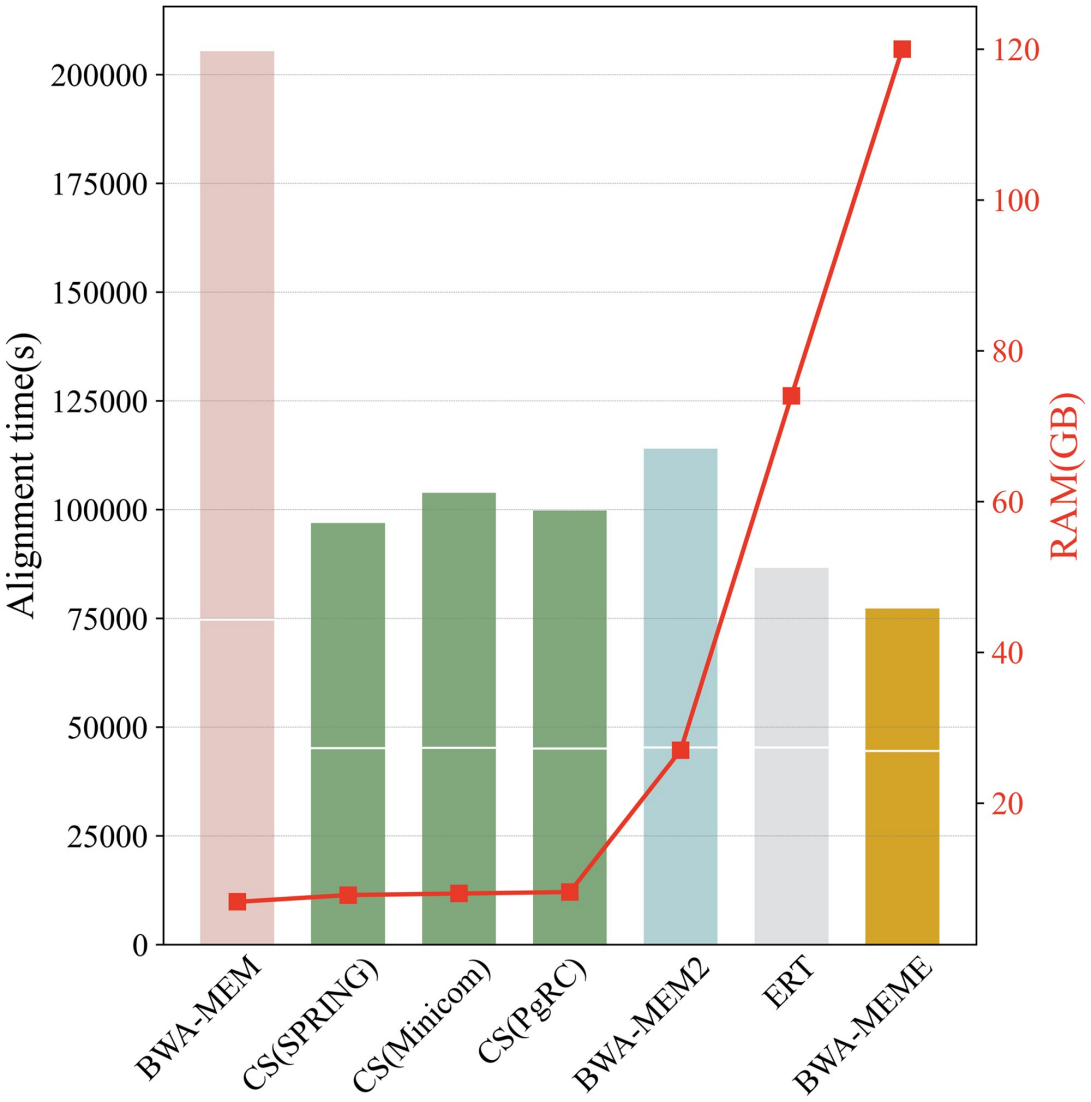
The alignment time and memory consumption of CompSeed (CS for short) and the existing optimization solutions. The white horizontal line in bars separates the seeding (above) and extension (below) time.

We also evaluated CompSeed on the dataset ERP001775 against other seeding optimization solutions, which are based on hardware programming and/or machine learning (Fig. 5). Those solutions replace the FM-index of BWA-MEM with a self-designed index structure, and exhaustively optimize every detail to improve the space locality of seeding. CompSeed does not offer speed advantage in comparison with those methods. But this does not diminish the value of CompSeed. First, CompSeed requires much less RAM memory (∼8 GB) compared with ERT and BWA-MEME. When dealing with larger scale reference genomes or pangenome collections, memory price would be an important consideration. Second, CompSeed achieves the acceleration by merging redundant operations in general seeding, and similar redundancy also exists in the existing optimization methods. We found that these methods are generally faster on reordered reads due to better spatial locality (Supplementary Table S10). Further improvement is promising if they explicitly incorporate the idea of CompSeed.

In the end, we compared the overall time cost of our compressive pipeline, which includes professional compression along with CompSeed alignment, to that of the traditional routine involving gzip compression and BWA-MEM alignment. As shown in Table 3, even when accounting for compression and decompression time, CompSeed with SPRING or PgRC remains faster than BWA-MEM with gzip. In such cases, the compressive pipeline clearly emerges as a superior choice, offering both space and time savings. Although CompSeed with Minicom results in an expensive pipeline due to the slow compression, users are allowed to choose efficient or preferred ones since CompSeed is compatible with any similar compressor. We anticipate that in the future, more practical tailored tools will be proposed, serving as the driving force behind compressive alignment and eventually replacing commonly used general-purpose compressors.

**Table 3.** The overall time (s) of compression, decompression, and alignment.

Accession No.	BWA-MEM	CompSeed with		
	with gzip	SPRING	Minicom	PgRC
SRR1562082	340	220	498	198
SRR16905161	3919	3479	16 613	2629
SRR13537343	65 059	42 044	150 561	39 558
ERP001775	222 813	139 132	561 415	123 840
ERR194146	197 357	148 715	519 175	128 500
ERR194161	195 793	146 006	499 845	120 613
ERR3239279	143 030	123 968	949 849	104 189
SRR10965089	165 603	133 167	1 077 333	134 239

## 4 Conclusion and discussion

In this study, we introduce the compressive seeding algorithm, CompSeed, as a solution to accelerate the BWA-MEM seeding, which has been the major bottleneck in sequence alignment. In the current scenario, where sequencing data are compressed by the effective reordering-based methods for saving storage price, CompSeed is able to collaborate with these compressors and directly leverage the information from the compressed sequences. In the reordered layout of reads, the intermedia results of preceding reads are stored in the SST and reused by subsequent reads, thereby avoiding a large number of repetitive BWT inquiries and SAL. By merging redundant computations, CompSeed is ∼2× faster than the BWA-MEM seeding. Additionally, CompSeed does not increase the memory consumption as it does not modify or replace the FM-index. The memory advantage of FM-index is preserved, and it is prominent when compared to the machine-learning-based solution.

In addition to reduce the computational cost of sequencing alignment, our study extends the significance of upstream compression. Although achieving absolutely superior compression ratios and gradually accepted in the genomic community, the upstream compression tools are overdue to replace the general-purpose tools like gzip. In our opinion, it is partly because the downstream aligners are not compatible with the specialized tools. We believe that making aligners compatible with and beneficial from the specialized compressors can promote the adoption of cost-effective tools and authorized compression standards.

Currently, CompSeed works on single-end reordered reads for demonstrating the feasibility and effectiveness of the compressive seeding algorithm. A complete tool incorporating functions of compression that enables paired-end alignment is our future work.

## Supplementary Material

btae100_Supplementary_Data

## Data Availability

The Accession Numbers in the main tables provide the necessary availability information for those public datasets. The downloadable links are presented in the Datasets section of Supplementary Materials.
