# Supplementary material for: A compressive seeding algorithm in conjunction with reordering-based compression
Source: Bioinformatics. 2024 Feb 20;40(3):btae100. doi: 10.1093/bioinformatics/btae100 (PMC10955252; doi:10.1093/bioinformatics/btae100)
Supplement: btae100_Supplementary_Data [file btae100_supplementary_data.docx]

A compressive seeding algorithm in conjunction with reordering-based compression (Supplementary Materials)

Fahu Ji, Qian Zhou, Jue Ruan, Zexuan Zhu, Xianming Liu

# Datasets

Table S1. Reference sequences availability.

| *E.* coli | <https://ftp.ncbi.nlm.nih.gov/genomes/all/GCF/000/005/845/GCF_000005845.2_ASM584v2/GCF_000005845.2_ASM584v2_genomic.fna.gz> |
| --- | --- |
| *C.* elegans | <https://ftp.ncbi.nlm.nih.gov/genomes/all/GCF/000/002/985/GCF_000002985.6_WBcel235/GCF_000002985.6_WBcel235_genomic.fna.gz> |
| *G*. gallus | <https://ftp.ncbi.nlm.nih.gov/genomes/all/GCF/016/699/485/GCF_016699485.2_bGalGal1.mat.broiler.GRCg7b/GCF_016699485.2_bGalGal1.mat.broiler.GRCg7b_genomic.fna.gz> |
| *H*. sapiens  (GRCh37) | <https://ftp.ncbi.nlm.nih.gov/genomes/all/GCF/000/001/405/GCF_000001405.25_GRCh37.p13/GCF_000001405.25_GRCh37.p13_genomic.fna.gz> |

Table S2. Sequencing data availability.

| SRR1562082 | <ftp://ftp.sra.ebi.ac.uk/vol1/fastq/SRR156/002/SRR1562082/SRR1562082_1.fastq.gz> |
| --- | --- |
| SRR16905161 | <ftp://ftp.sra.ebi.ac.uk/vol1/fastq/SRR169/061/SRR16905161/SRR16905161.fastq.gz> |
| SRR13537343 | <ftp://ftp.sra.ebi.ac.uk/vol1/fastq/SRR135/043/SRR13537343/SRR13537343_1.fastq.gz> |
| ERP001775  (7X, 14X, 21X, 28X, 35X) | <ftp://ftp.sra.ebi.ac.uk/vol1/fastq/ERR174/ERR174324/ERR174324_1.fastq.gz>  <ftp://ftp.sra.ebi.ac.uk/vol1/fastq/ERR174/ERR174325/ERR174325_1.fastq.gz>  <ftp://ftp.sra.ebi.ac.uk/vol1/fastq/ERR174/ERR174326/ERR174326_1.fastq.gz>  <ftp://ftp.sra.ebi.ac.uk/vol1/fastq/ERR174/ERR174327/ERR174327_1.fastq.gz>  <ftp://ftp.sra.ebi.ac.uk/vol1/fastq/ERR174/ERR174328/ERR174328_1.fastq.gz> |
| ERR194146 | <ftp://ftp.sra.ebi.ac.uk/vol1/fastq/ERR194/ERR194146/ERR194146_1.fastq.gz> |
| ERR194161 | <ftp://ftp.sra.ebi.ac.uk/vol1/fastq/ERR194/ERR194161/ERR194161_1.fastq.gz> |
| ERR3239279 | <ftp://ftp.sra.ebi.ac.uk/vol1/fastq/ERR323/009/ERR3239279/ERR3239279_1.fastq.gz> |
| SRR10965089 | <ftp://ftp.sra.ebi.ac.uk/vol1/fastq/SRR109/089/SRR10965089/SRR10965089.fastq.gz> |

Table S3. Detailed sequencing information.

| SRA ID | No. of Reads | Length | Coverage | Sequencing Platform |
| --- | --- | --- | --- | --- |
| SRR1562082 | 5,825,771 | 101 | 127X | Illumina HiSeq 2500 |
| SRR16905161 | 15,046,618 | 151 | 23X | Illumina HiSeq 4000 |
| SRR13537343 | 201,583,321 | 125 | 24X | Illumina HiSeq 2500 |
| ERP001775 | 863,941,088 | 101 | 28X | Illumina HiSeq 2000 |
| ERR194146 | 813,180,578 | 101 | 27X | Illumina HiSeq 2000 |
| ERR194161 | 843,454,257 | 101 | 28X | Illumina HiSeq 2000 |
| ERR3239279 | 420,210,145 | 150 | 21X | Illumina NovaSeq 6000 |
| SRR10965089 | 376,183,716 | 150 | 19X | BGISEQ-500 |

# Commands

**Compression and decompression**

SPRING (<https://github.com/shubhamchandak94/SPRING>)

spring -c -t16 --no-ids --no-quality -r -i in.fq -o out.spring

spring -d -i out.spring -o spring.reads

Minicom (<https://github.com/yuansliu/minicom>)

minicom -r in.fq -t16

minicom -d in_comp.minicom

PgRC (<https://github.com/kowallus/PgRC>)

pgrc -t16 -i in.fq out.pgrc

pgrc -d out.pgrc

**FM-Index building** (<https://github.com/lh3/bwa>)

bwa index –p ref ref.fa

**Alignment** (<https://github.com/i-xiaohu/CompSeed>)

CompSeed -t16 ref input.reads

bwamem -t16 ref input.reads

Note: bwamem in our repository comes from bwa release v0.7.17 with time trackers inserted to profile seeding and extension time.

# Additional results

The compression ratio and compression resource consumptions for reads are listed in Table S4 and S5, respectively. SPRING and PgRC demonstrate their practicality with much higher compression ratio for reads and acceptable time/RAM consumption in comparison with general-purpose tool gzip, while Minicom has an overly high memory consumption. All compression tools were run with 16 threads, pigz was used to enable multi-threading of gzip, and CPU time was recorded.

Table S4. Compression size for reads.

| Dataset | Reads | SPRING | Minicom | PgRC | gzip |
| --- | --- | --- | --- | --- | --- |
| SRR1562082 | 594 MB | 6 MB | 7 MB | 5 MB | 176 MB |
| SRR16905161 | 2.3 GB | 77 MB | 82 MB | 57 MB | 641 MB |
| SRR13537343 | 25.4 GB | 590 MB | 784 MB | 546 MB | 7.5 GB |
| ERP001775 | 88.1 GB | 1.7 GB | 2.4 GB | 1.5 GB | 26.1 GB |
| ERR194146 | 82.9 GB | 2.3 GB | 2.4 GB | 1.5 GB | 6.6 GB |
| ERR194161 | 86.0 GB | 2.2 GB | 2.3 GB | 1.5 GB | 6.7 GB |
| ERR3239279 | 63.5 GB | 1.5 GB | 1.9 GB | 1.2 GB | 4.4 GB |
| SRR10965089 | 56.8 GB | 1.7 GB | 2.2 GB | 1.6 GB | 16.7 GB |

Table S5. Compression runtime and memory consumption for reads.

| Dataset | Compression Runtime (s) | | | | Compression Peak RAM | | | |
| --- | --- | --- | --- | --- | --- | --- | --- | --- |
|  | SPRING | Minicom | PgRC | gzip | SPRING | Minicom | PgRC | gzip |
| SRR1562082 | 109 | 328 | 101 | 123 | 1.8 GB | 2.2 GB | 263 MB | 10 MB |
| SRR16905161 | 1655 | 14724 | 842 | 431 | 2.2 GB | 5.7 GB | 1.3 GB | 10 MB |
| SRR13537343 | 10530 | 118194 | 8485 | 5077 | 10.4 GB | 52.2 GB | 14.2 GB | 11 MB |
| ERP001775 | 40399 | 455281 | 23792 | 17413 | 42.6 GB | 185.0 GB | 40.0 GB | 11 MB |
| ERR194146 | 45731 | 411345 | 26906 | 3382 | 39.8 GB | 178.0 GB | 39.6 GB | 11 MB |
| ERR194161 | 45018 | 393784 | 22066 | 3796 | 41.6 GB | 182.3 GB | 39.8 GB | 11 MB |
| ERR3239279 | 45148 | 865464 | 23672 | 2568 | 23.9 GB | 122.3 GB | 38.1 GB | 10 MB |
| SRR10965089 | 40715 | 978846 | 39087 | 11314 | 21.7 GB | 114.7 GB | 35.2 GB | 11 MB |

As for decompression resource consumption (Table S6), both SPRING and Mincom have a reasonable decompression speed, indicating a potential seamless ‘pipe’ to feed input to aligners. PgRC is faster than gzip, though it has a higher memory usage. All tools were run in 16 threads, except for PgRC that is single-threaded in decompression mode.

Table S6. Decompression runtime and memory consumption for reads.

| Dataset | Decompression Runtime (s) | | | | Decompression Peak RAM | | | |
| --- | --- | --- | --- | --- | --- | --- | --- | --- |
|  | SPRING | Minicom | PgRC | gzip | SPRING | Minicom | PgRC | gzip |
| SRR1562082 | 16 | 66 | 2 | 4 | 767 MB | 86 MB | 160 MB | 700 KB |
| SRR16905161 | 59 | 125 | 9 | 16 | 1.6 GB | 332 MB | 650 MB | 704 KB |
| SRR13537343 | 996 | 677 | 77 | 173 | 2.6 GB | 2.9 GB | 6.6 GB | 704 KB |
| ERP001775 | 1812 | 2254 | 292 | 593 | 5.5 GB | 5.3 GB | 17.9 GB | 708 KB |
| ERR194146 | 2101 | 2188 | 238 | 404 | 6.5 GB | 5.3 GB | 17.8 GB | 708 KB |
| ERR194161 | 2011 | 2257 | 232 | 407 | 7.2 GB | 5.3 GB | 17.7 GB | 704 KB |
| ERR3239279 | 1154 | 1634 | 188 | 299 | 6.7 GB | 5.1 GB | 16.4 GB | 704 KB |
| SRR10965089 | 1174 | 1680 | 229 | 398 | 6.6 GB | 5.1 GB | 15.8 GB | 708 KB |

SPRING was run with the options ‘-r’ (allowing read reordering) and ‘-q ill_bin’ (lossy compression for quality scores to achieve a better compression ratio without compromising downstream analysis), and showed a compelling advantage in compression ratio compared to gzip (Table S7). Although gzip is more time- and memory-efficient, the compression and decompression performance (Table S8) of SPRING are good enough for typical use, considering its substantial space-saving capability.

Table S7. Compression performance for FASTQ files.

| Dataset | FASTQ | Size | | Runtime (s) | | Peak RAM | |
| --- | --- | --- | --- | --- | --- | --- | --- |
|  |  | SPRING | gzip | SPRING | gzip | SPRING | gzip |
| SRR1562082 | 1.6 GB | 113 MB | 472 MB | 252 | 181 | 2.2 GB | 11 MB |
| SRR16905161 | 5.6 GB | 448 MB | 1.3 GB | 1944 | 600 | 4.4 GB | 10 MB |
| SRR13537343 | 62.0 GB | 3.0 GB | 14.9 GB | 12712 | 6520 | 11.5 GB | 11 MB |
| ERP001775 | 223.3 GB | 19.6 GB | 71.7 GB | 60413 | 26782 | 42.8 GB | 11 MB |
| ERR194146 | 219.5 GB | 8.2 GB | 55.7 GB | 69198 | 12396 | 40.0 GB | 11 MB |
| ERR194161 | 227.2 GB | 7.5 GB | 54.0 GB | 64620 | 12755 | 41.8 GB | 11 MB |
| ERR3239279 | 153.9 GB | 7.7 GB | 14.2 GB | 46440 | 4849 | 24.0 GB | 10 MB |
| SRR10965089 | 127.3 GB | 12.6 GB | 48.0 GB | 52682 | 18701 | 21.9 GB | 11 MB |

Table S8. Decompression performance for FASTQ files.

| Dataset | Runtime (s) | | Peak RAM | |
| --- | --- | --- | --- | --- |
|  | SPRING | gzip | SPRING | gzip |
| SRR1562082 | 82 | 12 | 2.8 GB | 1 MB |
| SRR16905161 | 269 | 37 | 5.3 GB | 1 MB |
| SRR13537343 | 2950 | 410 | 5.7 GB | 1 MB |
| ERP001775 | 13754 | 1597 | 6.7 GB | 1 MB |
| ERR194146 | 13218 | 1517 | 6.5 GB | 1 MB |
| ERR194161 | 13136 | 1500 | 7.4 GB | 1 MB |
| ERR3239279 | 7772 | 706 | 8.0 GB | 1 MB |
| SRR10965089 | 6683 | 1008 | 8.1 GB | 1 MB |

Table S9. Resource consumption for building indexes.

| Genome | Runtime (s) | | | | Peak RAM (GB) | | | |
| --- | --- | --- | --- | --- | --- | --- | --- | --- |
|  | FM-index | MEM2 | ERT | MEME | FM-index | MEM2 | ERT | MEME |
| E. coli | 2 | 3 | 1902 | 55 | 0.01 | 0.1 | 24.1 | 2.8 |
| C. elegans | 71 | 78 | 3939 | 913 | 0.1 | 2.2 | 24.4 | 12.6 |
| G. gallus | 1167 | 1086 | 21579 | 7719 | 1.5 | 23.5 | 26.5 | 60.3 |
| H. sapiens | 3474 | 1656 | 60998 | 24506 | 4.4 | 70.2 | 32.1 | 96.5 |

We also tested the hardware optimizations, namely BWA-MEM2, ERT and BWA-MEME, on reordered reads, and found that reordering can improve their performance in varying degree due to better spatial locality. We assume that these professional tools could be further improved if they explicitly handle the data redundancy like CompSeed does. The reason that BWA-MEM2, ERT and BWA-MEME increasingly request for more memory space is to break through the memory bandwidth restriction, which is fully discussed in the publication of ERT. Removing data redundancy means fewer data is required to be fetched, therefore the data efficiency could be further improved.

Table S10. Seeding time of BWA-MEM2, ERT, BWA-MEME on reordered reads.

| Dataset | Aligner | FASTQ | Reordered by | | |
| --- | --- | --- | --- | --- | --- |
|  |  |  | SPRING | Minicom | PgRC |
| ERP001775 | BWA-MEM2 | 68752 | 35408 | 39984 | 39568 |
|  | ERT | 41344 | 28800 | 25600 | 26560 |
|  | BWA-MEME | 32752 | 20352 | 21392 | 21520 |
| ERR194146 | BWA-MEM2 | 37376 | 35568 | 38768 | 38560 |
|  | ERT | 25920 | 25904 | 24752 | 26096 |
|  | BWA-MEME | 21328 | 19904 | 18400 | 21440 |
| ERR194161 | BWA-MEM2 | 36928 | 35152 | 38752 | 39248 |
|  | ERT | 25872 | 24544 | 24848 | 24864 |
|  | BWA-MEME | 20592 | 17584 | 20528 | 20560 |
| ERR3239279 | BWA-MEM2 | 27520 | 26272 | segfault | 30112 |
|  | ERT | 16544 | 17328 | 16992 | 17040 |
|  | BWA-MEME | 15056 | 14768 | 15232 | 15712 |
| SRR10965089 | BWA-MEM2 | 46928 | 29920 | 31776 | 32048 |
|  | ERT | 21184 | 17152 | 17856 | 18080 |
|  | BWA-MEME | 24544 | 17600 | 18272 | 18416 |

To ensure that the benefit from CompSeed is indeed due to the reordered reads and not due to a better implementation of seeding, we also share results for CompSeed applied on the original FASTQ without any reordering. As the Table S11 shown, on most of datasets, CompSeed was slower than BWA-MEM seeding because there were few BWT queries and SAL to merge for randomly ordered reads. The occasional hits in SST did not offset the overhead of SST, especially when the tries were frequently branching and led to more cache misses. We specifically checked the outliers of ERR194146, ERR194161 and ERR3239279, found that they were reordered/clustered (not thoroughly) in prior gzip compression. Researchers had long realized reordering can improve the compression ratio of general-purpose tools. The three sequencing datasets are all relatively new, indicating the professional tools are already assisting - though not fully replacing - the traditional compression. That explains why CompSeed showed a not complete speedup on them. Nevertheless, it does not harm the conclusion that the speed advantage of CompSeed sources from reordering. We can infer that from the reduction of BWT queries and SAL. Only reordered reads provide such opportunity to merge those repetitive computations.

Table S11: CompSeed on original FASTQ files.

| Dataset | Seeding runtime (s) | | BWT queries per read | | SAL per read | |
| --- | --- | --- | --- | --- | --- | --- |
|  | BWA | CompSeed | BWA | CompSeed | BWA | CompSeed |
| SRR1562082 | 165 | 168 | 314 | 240 | 6.54 | 5.47 |
| SRR16905161 | 1529 | 1713 | 716 | 503 | 22.09 | 17.00 |
| SRR13537343 | 28849 | 30919 | 712 | 527 | 28.97 | 21.33 |
| ERP001775 | 150725 | 160263 | 619 | 475 | 29.00 | 23.03 |
| ERR194146 | 121849 | 71412 | 629 | 270 | 29.62 | 14.42 |
| ERR194161 | 122252 | 70562 | 613 | 261 | 28.71 | 13.81 |
| ERR3239279 | 86982 | 52276 | 900 | 386 | 38.75 | 17.70 |
| SRR10965089 | 98605 | 110289 | 998 | 740 | 40.92 | 31.77 |
